# Truncated Core/NS3 Fusion Protein of HCV Adjuvanted with Outer Membrane Vesicles of *Neisseria meningitidis* Serogroup B: Potent Inducer of the Murine Immune System

**DOI:** 10.29252/.23.4.235

**Published:** 2019-07

**Authors:** Soheila Hekmat, Seyed Mehdi Sadat, Mohammad Mehdi Aslani, Mehdi Mahdavi, Azam Bolhassani, Fateme Asgar Halvaee, Seyed Mohammad Mahdi Ghahari, Mohammad Reza Aghasadeghi, Seyed Davar Siadat

**Affiliations:** 1 Department of Hepatitis and AIDs, Pasteur Institute of Iran, Tehran, Iran; 2 Department of Microbiology, Pasteur Institute of Iran, Tehran, Iran; 3 Recombinant Vaccine Research Center, Tehran University of Medical Sciences, Tehran, Iran; 4 Department of Mycobacteriology and Pulmonary Research, Pasteur Institute of Iran, Tehran, Iran

**Keywords:** Adjuvants, Hepatitis C virus, Immunization, Vaccines

## Abstract

**Background:**

A licensed vaccine against hepatitis C virus (HCV) has not become available to date. The stability and antigenicity of a targeted synthesized recombinant fusion protein consisting of a truncated core and NS3 (rC/N) of HCV had been predicted. Although safe antigens, recombinant proteins are not efficacious vaccines without adjuvants. The present study evaluated the immunogenicity of rC/N as a bipartite antigen accompanied by *Neisseria meningitidis* serogroup B outer membrane vesicles (*NM*B OMVs) in BALB/c mice.

**Methods:**

The *NM*B OMVs were produced and evaluated accurately. The administrations were as follows: rC/N-OMV, rC/N-Freund’s complete/incomplete adjuvant (CIA), rC/N-MF59, rC/N, OMV, MF59, and PBS. The production of Th1 (IFN-γ, IL-2)/Th2 (IL-4)/Th17 (IL-17) cytokines and granzyme B (cytotoxic indicator) by splenic mononuclear cells and the humoral concentration of total IgG/IgG1 (Th2)/IgG2a (Th1) in sera of mice were measured using mouse ELISA kits.

**Results:**

Concentrations of Th1/Th2/Th17 cytokines, granzyme B, and immunoglobulins in the spleens and sera of immunized mice, which had received antigen plus each adjuvant (rC/N-OMV, rC/N-Freund’s CIA, and rC/N-MF59), significantly raised compared to the controls (rC/N, OMV, MF59, and PBS). Th1-type responses were dominant over Th2-type responses in vaccinated mice with rC/N-OMV, and Th2 type responses increased dominantly in vaccinated mice with rC/N-MF59 (*p* < 0.05).

**Conclusion:**

*NM*B OMVs were able to increase Th1 immune responses dramatically more than MF59 and Freund’s CIA. The formulation of rC/N with *NM*B OMVs showed its ability to induce Th1, Th2, and Th17 immune responses. rC/N-*NM*B OMVs is a promising approach for the development of an HCV therapeutic vaccine.

## INTRODUCTION

Approximately, 71 million people around the world are living with chronic hepatitis C virus (HCV) infection. Some complications such as cirrhosis and hepatocellular carcinoma (HCC) are more likely to occur in a significant number of individuals with this disease. Around 399,000 people suffering from HCV die each year. Although HCV is a curable disease, only 1.5% of infected patients have access to medication[1]. Currently, there is no effective and licensed vaccine available to provide treatment or protection against HCV infection[2]. In about 25% of HCV patients, the virus is spontaneously self-limiting after elevations in CD8^+^ cytotoxic T lymphocytes (CTLs), CD4^+^ T helper cells (Th cells), and neutralizing antibodies. Therefore, an effective vaccine against chronic HCV infection may be due to the balance between cellular-mediated and humoral-mediated immune responses[3,4].

One of the most important targets of HCV therapeutic vaccines is the prevention of chronicity of infection and the prevention of cell damage (cirrhosis and HCC) by increased cellular immune responses. Despite the preference of DNA vaccine to induce Th1 immune responses, this type of vaccination has a risk of potentially disrupting normal cellular processes. This is a major concern that the introduction of foreign DNA into the host cells could affect normal protein expression pathways of the cell. Thus, fusion protein- and peptide-based vaccines are safer than DNA- and viral vector-based vaccine for the prevention of cirrhosis and HCC[5-7].

Structural [core, envelope (E) 1 and 2, p7] and non-structural [(NS)2, NS3, NS4A, NS4B, NS5A and NS5B] proteins of HCV have been considered as candidate antigens for inclusion in a multicomponent and Th1/Th2-oriented vaccine[7,8]. Core, NS3, and NS4 were specially under focus to develop therapeutic vaccine against HCV (Okairos, IC41, Inter cell AG) and completed phases I and II of clinical trial[6,7]. Core and NS3 are both the most conserved proteins of HCV. Due to the induction of CTLs and Th cells, the humoral immune activity and eventual clearance of the virus by the immune system have been considered in various therapeutic vaccine development studies[9-14]. However, the full-length NS3 protein has serine protease and RNA helicase enzymatic activities, and it has immunosuppressiveeffects on antigen presenting cells, which are noxious to the host[12-14]. Furthermore, the full-length core protein has autoimmune activity, which may be harmful to humans and results in immune suppression/modulation in animal models[9-11,13].

Three adenoviral constructs expressing the middle region of the core gene and consisting of some parts of D1 and D2 (aa 50-160) and the middle region of NS3 consisting of protease and helicase (aa 1095-1387) have been developed by Hosseini *et al*.[15,16]. In order to retain the conformational structure and more immune response of the recombinant core, the full-length of core D1 (aa 1-118) with a flexible linker (AAY) and proteasome cleavable site and also the middle region of NS3 in fused form (rC/N) were produced without C-terminal domains of core, and without N and C-terminal sequences of NS3. As a result, the harmful activities of core and also enzymatic activities of the full-length of NS3 will be eliminated[17]. In this regard, the bipartite-truncated recombinant protein (rC/N), as a new vaccine candidate, was analyzed using bioinformatic software, synthesized and carefully evaluated[17].

Although the recombinant protein vaccines are safe and purposeful, such vaccines typically evokes weak immunity. Hence it is necessary to include one or more immune-stimulatory components in the vaccine formulation to induce potent, targeted and prolonged immune responses against the administered antigen[18,19]. Most of the substances constituting a major potential source of adjuvants are unable to receive FDA approval for use in human vaccine formulations. Thus, there is an essential need for safer, non-toxic and more effective adjuvants, especially to stimulate both Th1 and Th2 immune responses against co-inoculated antigens for protection against many pathogens[20-22]. Among licensed adjuvants, those with microbial origin are more notable[21]. The outer membrane vesicles (OMVs) are naturally non-replicating and highly immunogenic, mostly derived from Gram-negative bacteria. Detergent-generated vesicles are safe and able to trigger the production of numerous pro-inflammatory cytokines[23-25]. The vesicles are spherical nanoparticles (50-300 nm in diameter) and consist of phospholipids, lipopolysaccharide (LPS), outer membrane proteins (OMPs), and entrapped periplasmic components of their parents[26,27]. *Neisseria meningitidis* serogroup B OMVs (*NM*B OMVs) have shown evidence of potency and safety, even as a vaccine in infants, against meningococcal meningitis[27-29] and also as an adjuvant to promote cellular and humoral immune responses in a broad range of viral and bacterial vaccines[26,30-32]. The OMVs of *Escherichia coli* are also used as an adjuvant and delivery system for cancer immunotherapy in mice[33].

The present study aimed to prescribe concurrently the *NM*B OMVs, as an adjuvant, with the truncated recombinant core_1-118_ (rCore) and NS3_1095-1384_ (rNS3) fusion protein (rC/N) of HCV to evaluate the probable immune responses against HCV in a murine model.

## MATERIALS AND METHODS

### rC/N fusion protein production

The rC/N fusion protein was previously prepared[17]. Briefly, the first domain of the core (amino acid residues 1-118), an AAY linker, and the middle region of NS3 (aa 1095-1384) were cloned into the pET 24a (+) vector, expressed in *E. coli* BL21-DE3, purified by affinity chromatography and finally analyzed by SDS-PAGE and Western blotting using anti-6× His tag monoclonal antibody.

### NMB OMVs preparation and analysis

*NM*B OMVs were prepared as described before[26]. In brief, the *NM*B strain (CSBPI, G-245) was grown under controlled submerged cultural conditions in a fermentor containing modified Frantz medium at 36 ± 1 °C for 24 h, up to the early stationary phase. OMVs were released into 0.1 M of Tris-HCl buffer, pH 8.6, containing 10 mM EDTA and 0.5% w/v sodium deoxycholate. Purification of the OMVs was done by centrifugation at 20,000 ×g for 30 minutes, followed by ultracentrifugation at 125,000 ×g for 2 h. The sediment of vesicles was homogenized in PBS, pH 7.2, and thimerosal (100 mg/l) was added as a preservative.

### Analysis of NMB OMVs

Total protein content of OMVs was measured by spectrophotometry. The protein concentration was also determined using the Bradford assay. The POR A protein of *NM*B OMVs was analyzed on 12% SDS-PAGE gel. The LPS amount in the *NM*B OMVs was assayed using the Limulus Amebocyte Lysate (LAL) method (Cambrex Corporation, USA), and the pyrogenicity of the OMVs was assayed in rabbit. Briefly, three out of four healthy adult New Zealand Albino rabbits, weighing between 1.8 and 3.7 kg, were injected (10 ml/per kg body weight) with *NM*B OMV. One rabbit was considered as the control. The rectal temperatures of each rabbit were measured at 30-minute intervals between 1-3 hours subsequent to the injection[28]

### Morphology of NMB OMVs

An Atomic Frorce Microscope (AFM, Nano Wizard II nano-science AFM, JPK Instruments Inc., Germany) and JPK data processing software were used to observe the morphology of *NM*B OMVs. Mean size distribution, polydispersity index (PDI), and zeta potentials of the *NM*B OMVs were determined by dynamic light scattering (Zetasizer Nano ZS; Malvern Instruments, Malvern, UK) at 25 °C.

### Antigen formulation

The rC/N protein alone and admixed forms (rC/N-OMV, rC/N-Freunds CIA, and rC/N-MF59) were prepared for immunization. Each dose contained 20 µg of rC/N admixed with 40 µg of adjuvant in a total volume of 100 µl sterile PBS and was shaken vigorously.

### Experimental groups and immunization schedule

Pathogen-free 6-8-week-old female BALB/c mice were divided into seven groups, each consisting of six animals housed according to international animal care ethics and immunized subcutaneously with rC/N, rC/N-OMV, rC/N-Freund’s CIA, and rC/N-MF59. The MF59, OMV, and PBS were used as controls. Each mouse received 20 µg rC/N alone or emulsified in 40 µg of each adjuvant in a total volume of 100 µl. Booster injections were given three and six weeks after the first prescriptions.

### Total antibody and isotypes assay

Blood was collected from immunized mice by retro-orbital bleeding two weeks after the third immunization, and the sera were stored at -20 °C until use. An ELISA was used to determine humoral responses. Briefly, 1 μg/ml of rC/N or rCore or rNS3 protein in PBS was added to each well of 96-well ELISA Maxisorp plates (Nunc, Naperville, IL, USA) and incubated at 4 °C overnight. Plates were washed with washing buffer (PBS containing 0.05% Tween 20) and blocked at 37 °C for 1 h using the blocking buffer (PBS containing 0.05% skimmed milk). The wells were washed, and serial dilutions of sera from 1:50 to 1:102400 were added to each well and incubated at 37 °C for 1.5 h. Then the wells were washed and incubated with 1:10,000 dilution of anti-mouse antibody HRP conjugate (Sigma, USA) for 1.5 h. The washing step was repeated, and the plate was incubated with TMB substrate in the dark for 30 min. The reaction was stopped with 2N H_2_SO_4_, and color density was measured at OD_450_ nm. The specific IgG1 and IgG2a subclasses were detected using goat anti-mouse IgG1 and IgG2a secondary antibodies (Sigma, USA) according to the manufacturer’s instructions.

### Cytokine assay

Three weeks after the last booster injection, the spleen of each mouse was dissected under sterile conditions, crushed and suspended in cold PBS containing 2% fetal bovine serum. Red blood cells were lysed, and a total number of 2 × 10^5^ splenocytes in 100 μl suspension were seeded on each well of a 24-well plate using complete RPMI 1640 (Gibco, Germany), supplemented with 10% FBS and 4 mM of L-glutamine and stimulated *in vitro* with 10 μg rC/N incubated in 5% CO_2_ at 37 °C for 72 h. The supernatants were collected and stored at -70 °C util use. The concentrations of IFN-γ, IL-4, IL-2, and IL-17 cytokines were estimated using ELISA Kits (e-Bioscience, San Diego, CA, USA) according to the manufacturer’s instruction. The concentration of each sample was calculated according to the standard curve.

### Granzyme B assay

The amount of granzyme B in the supernatant of cultured spleen cells was measured according to the Mouse Granzyme B ELISA kit instructions (Invitrogen by Thermo Fisher Scientific, Affymetrix, USA) using a quantitative sandwich ELISA assay. Several dilutions of granzyme B were used to adjust a calibration curve for determination of accurate granzyme B concentration in the sample. All wells were stimulated by rC/N, and few wells belonged to OMV-rC/N immunized group were prepared by antigen without any stimulation. Samples and standards were assayed at an optical density (OD) of 450 nm.

### Statistical analysis

The graphPad Prism 6.01 software was employed to evaluate differences between the experimental groups using one-way and two-way analysis of variance (ANOVA), followed by Tukey’s or Holm-Sidak’s multiple comparisons tests. Values of *p* < 0.05 were considered statistically significant.

## RESULTS

### Confirmation and safety of NMB OMVs

**Fig. 1:**
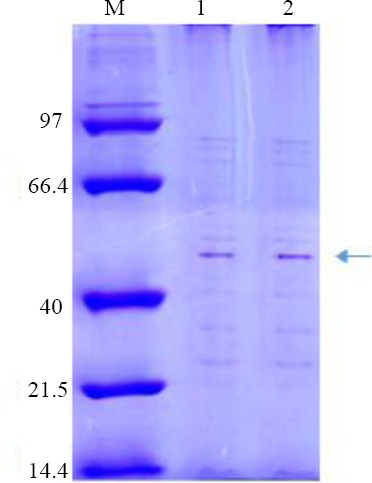
Confirmation of the *NM*B OMVs by SDS-PAGE. Lines 1-2, *NM*B OMVs; M, marker (14.4-97 kDa); Por A protein belonging to *NM*B OMP confirmed by a dominant 45-KDa bond in the SDS-PAGE pattern.

**Fig. 2:**
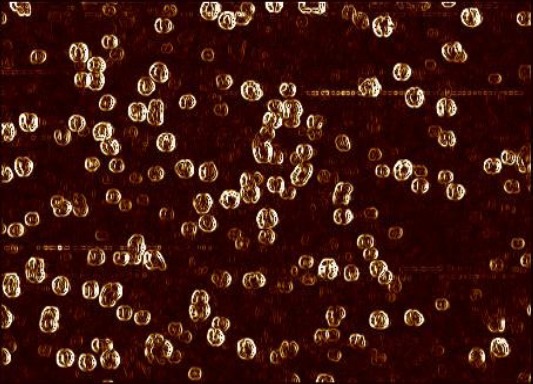
Atomic Force Microscopic (AFM) presentation of *NM*B OMVs. The selected channel to process was error signal trace mode (fast 1.788 × slow 1.152 μm).

**Fig. 3:**
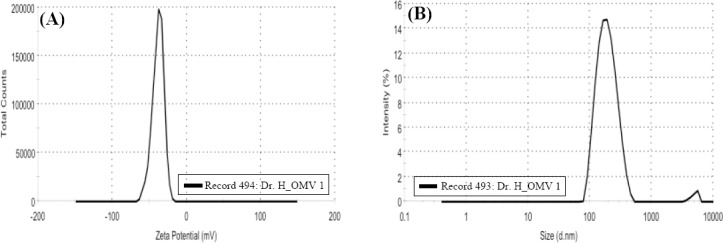
Dynamic light scattering analysis of *NM*B OMVs. (A) Zeta potential distribution of *NM*B OMVs; (B) Size distribution by intensity or zeta average and PDI.

The protein content of OMVs determined by spectrophotometry was 1.2 mg/ml, and the SDS-PAGE pattern confirmed the presence of POR A of *NM*B OMVs as class one OMP with a 45-kDa band (Fig. 1). The LAL test showed 0.8 EU/ml endotoxin activity. No rabbits showed individual temperature rises of 0.6 °C, and by utilizing *NM*B OMVs, the test met the requirements for the absence of pyrogeny. AFM scanning showed the uniformity and spherical morphology of the OMVs (Fig. 2). The absolute value of *NM*B OMVs’ zeta potential was 38.1 (| -38.1 | = 38.1 mV; Fig. 3A), and their zeta average size was 184.2 (d. nm; Fig. 3B). The heterogeneity index or PDI of vesicles was 0.178 (Fig. 3B).

### Humoral immune responses and isotyping

**Fig. 4:**
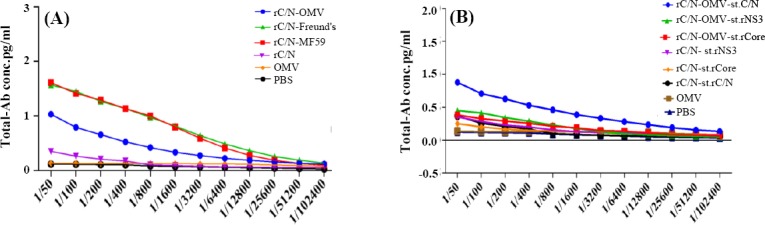
Total antibody in immunized mice. (A) Total antibody in mice immunized with candidate rC/N-OMV vaccine, rC/N-MF59, rC/N-Freund’s CIA, rC/N, OMV, and PBS after *in vitro* stimulation with rC/N protein. The rates of antibody were significantly higher in the vaccine groups than in the control groups (*p* < 0.05). The rates of total antibody in mice immunized with rC/N-MF59 and rC/N-Freund’s CIA were more than the vaccinated group with rC/N-OMV (*p* < 0.05); (B) total antibody in rC/N-OMV stimulated with rC/N was higher than rC/N-OMV stimulated with rCore, rNS3, or the controls (*p* < 0.05).

**Fig. 5:**
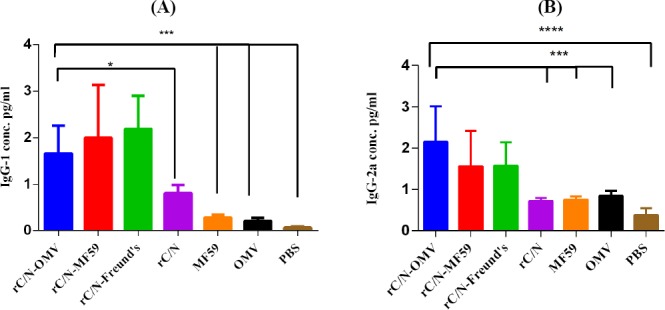
One-way ANOVA of specific humoral antibodies followed by Holm-Sidak’s multiple comparisons test. (A) The level of IgG1 in rC/N-OMV was significantly higher vs. rC/N (*p* < 0.05) and vs. MF59, OMV, and PBS (*p* < 0.0005). (B) the level of IgG2a in the candidate rC/N-OMV vaccine was significantly higher vs. rC/N (*p* < 0.0005), MF59 (*p* = 0.0003), OMV (*p* = 0.0004), and PBS (*p* = 0.0001). The levels of significancy are indicated by stars as follows: ^*^p < 0.05, ^***^p < 0.0005, and ^****^p < 0.0001.

The results showed that immunizing mice with the candidate rC/N-OMV vaccine induced higher levels of total antibody in the experimental groups compared with the controls (PBS, OMV, and MF59; *p* < 0.05; Fig. 4A). The results of total immunoglobulin titration in mice immunized with rC/N-OMV showed that the rC/N fusion protein was able to induce specific humoral responses more than rCore or rNS3 alone or the controls (*p* < 0.05; Fig. 4B). The rate of IgG1 in rC/N-OMV showed significant differences versus rC/N (*p* < 0.05), MF59 (*p* = 0.0012), OMV (*p* = 0.0004), and PBS (*p* = 0.0004; Fig. 5A). There are significant differences between the rC/N-OMV and the control groups (MF59, OMV, and PBS) in the induction of IgG2a (*p* = 0.0003, 0.0004, and 0.0001, respectively) in the sera of mice (Fig. 5B). The measurements of the antibody isotypes showed that the IgG2a level rose in the sera of the mice that received rC/N accompanied by OMV more than other immunized mice groups (*p* < 0.05). The IgG2a isotype is an indicator for Th1/cellular response[34]. The ratio of IgG2a concentration in splenocytes of mice that received rC/N-OMV was higher than all other vaccinated and control groups. It seems that OMV, as an adjuvant, is able to induce an IgG2a response more than MF59 and Freund’s CIA (*p* < 0.05; Fig. 5B).

### Evaluation of IFN-γ, IL-2, IL-4, and IL-17

**Fig. 6:**
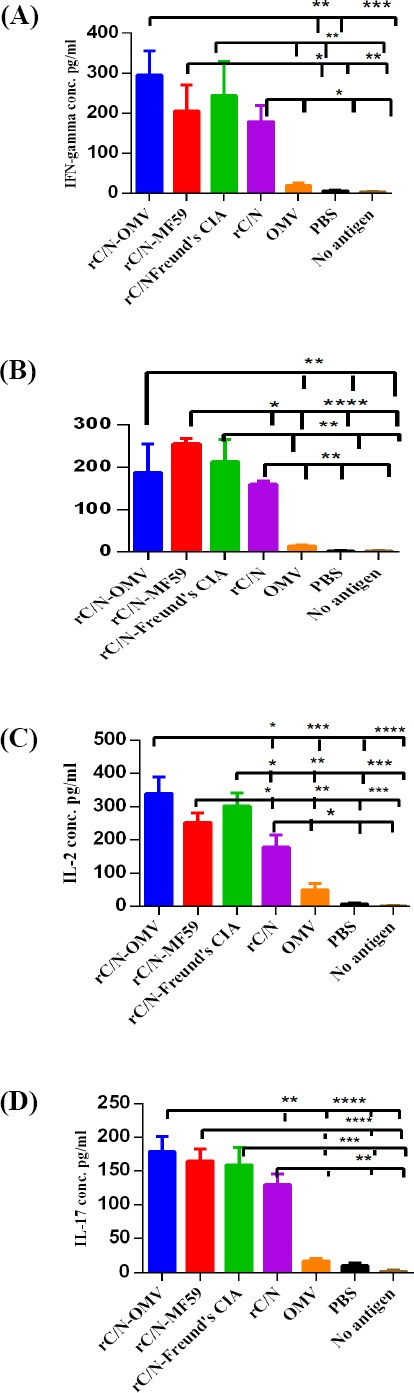
Cytokines secretion analysis of spleens in immunized mice after *in vitro* stimulation with rC/N protein and un-stimulated with any antigen. (A) IFN-γ, (B) IL-4, (C) IL-2, and (D) IL-17 cytokines secretion. The levels of significance are indicated by stars as follows: ^*^p < 0.05, ^**^p < 0.005, ^***^p < 0.0005, and ^****^p < 0.0001.

The concentrations of IFN-γ, IL-2, IL-4, and IL-17 cytokines from rC/N-stimulated mononuclear cells of spleens from mice groups vaccinated with or without adjuvant (rC/N, rC/N-OMV, rC/N-MF59, and rC/N-Freund’s CIA), and the controls were evaluated three weeks after the final booster injections. The results are shown in Figure 6.

As depicted in Figure 6A, the concentration of IFN-γ cytokine in the experimental groups after the injection of rC/N, rC/N adjuvanted with *NM*B OMVs, MF59 or Freund’s CIA significantly increased compared with the control groups (*p* < 0.05). No significant difference was observed among the control groups (*p* > 0.05). The rate of IFN-γ in mice immunized with rC/N-OMV differed significantly from the groups that received OMV (*p* = 0.001), PBS (*p* = 0.0007), or not stimulated with any antigen (*p* = 0.0006). The concentration of IFN-γ was significantly higher in the group of mice immunized with rC/N-MF59 than in the control groups (*p* < 0.005). The level of this cytokine in mice vaccinated with rC/N-Freund’s CIA differed significantly from that of the control groups (*p* < 0.005). The mice immunized with the rC/N were significantly different from the control groups (*p* < 0.05).

Immunization with all formulations of vaccines (rC/N-OMV, rC/N-MF59, and rC/N-Freund’s CIA) significantly increased IL-4 producing lymphocytes compared with the control groups (*p* < 0.005). The difference was greater between the rC/N-MF59 recipient group and the control groups for IL-4 than with other vaccinated groups (*p* < 0.0001). This group was significantly different from rC/N alone as well (*p* < 0.05). The intra-group comparison showed significant difference among rC/N-MF59 and other vaccine recipient groups (*p* > 0.05). Moreover, no significant intra-group difference was observed among the control groups (*p* > 0.05; Fig. 6. B).

The splenocytes from the immunized mice with vaccine groups (rC/N-OMV, rC/N-MF59, and rC/N-Freund’s CIA) showed significant proliferation and production of the IL-2 cytokine immune response compared with the control groups (*p* < 0.0005). The level of IL-2 was significantly different from the rC/N vaccinated group, as compared with the control groups (*p* < 0.05). The IL-2 concentration in all groups vaccinated with rC/N accompanied with adjuvants (OMV, MF59, and Freund’s CIA) was significantly different from that in the group receiving rC/N alone (*p* < 0.05; Fig. 6C).

As seen in Figure 6D, the pro-inflammatory IL-17 producing lymphocytes up-regulated significantly in the spleen of mice immunized with rC/N-OMV (*p* < 0.0001), rC/N-MF59 (*p* < 0.0005), rC/N-Freund’s CIA (*p* = 0.0005), and rC/N (*p* < 0.005) vs. OMV, PBS, and no stimulation with any antigen. The level of IL-17 was approximately equivalent in the groups immunized with rC/N-Freund’s CIA, rC/N-MF59, and rC/N-OMV (*p* > 0.05). Although the addition of adjuvants to rC/N protein increased the level of IL-17, no significant difference based on adjuvant type was observed.

### Comparison of cytokines

**Fig. 7:**
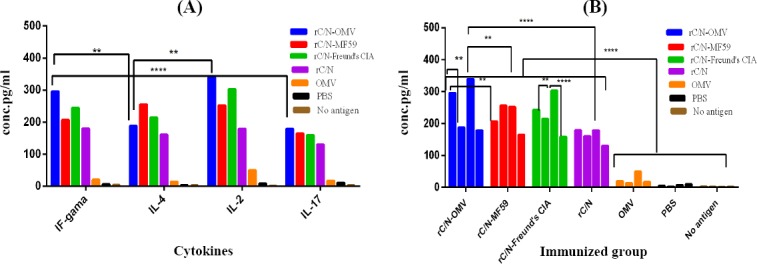
Comparison of cytokines (IFN-γ, IL-2, IL-4, and IL-17) in all immunized groups. (A) Comparison of Th1 (IFN-γ and IL-2), Th2 (IL-4), and Th17 (IL-17) cytokine secretion in the splenocytes of immunized mice groups (rC/N-OMV, rC/N-MF59, rC/N-Freund’s CIA, rC/N, OMV, PBS stimulated by rC/N, and no stimulated by antigen). The level of IFN-γ and IL-2 increased significantly compared with IL-4 and IL-17 (*p* < 0.005). (B) There was a significant difference between rC/N-OMV and rC/N-MF59 vaccinated groups in the secretion of IFN-γ and IL-2 cytokines (*p* < 0.005). The level of IL-2 was higher in splenocytes of mice immunized with rC/N-OMV than those immunized with rC/N (*p* < 0.0001). The levels of significance are indicated by stars as follows: ^**^p < 0.005, ^****^p < 0.0001.

The ratios of cytokines (IFN-γ, IL-2, IL-4, and IL-17) producing lymphocytes in the spleen of the immunized mice were compared with each other (Fig. 7). With respect to the comparative cytokines profile, the levels of the Th1 (IFN-γ and IL-2) immune response in immunized group with rC/N-OMV group was significantly higher than Th2 (IL-4) and Th17 (IL-17) immune responses (*p* < 0.05; Fig. 7A). The level of IFN-γ was significantly higher than IL-4 (*p* < 005) and IL-17 (*p* < 0.0001) in vaccinated mice group with the rC/N-OMV. The level of IFN-γ in spleen of vaccinated group with rC/N-OMV was significantly higher than the vaccinated mice group with rC/N-MF59 (*p* < 0.005) (Fig. 7B).

### Cytotoxic activity

**Fig. 8:**
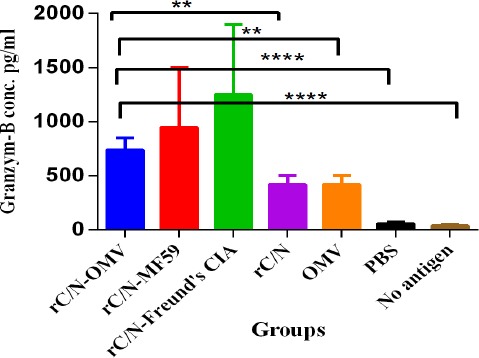
Cytotoxic activity in experimental groups. The level of granzyme B in the spleens of the mice immunized with the candidate rC/N-OMV vaccine increased significantly compared with the rC/N (*p* = 0.0015), OMV (*p* = 0.0015), PBS (*p* = 0.0001), and no antigen (*p* = 0.0001) groups. The levels of significance are indicated by stars as follows: ^**^p < 0.005, ^****^p < 0.0001.

The rate of granzyme B in the spleens of immunized mice was significantly higher in the vaccinated groups (rC/N-Freund’s CIA, rC/N-MF59, and rC/N-OMV) in comparison with the control groups [rC/N (*p* < 0.005), OMV (*p* < 0.005), PBS (*p* = 0.0001)] and no stimulation with any antigen (*p* = 0.0001), as shown in Figure 8. There was no significant difference between the groups vaccinated with rC/N-Freund’s CIA, rC/N-MF59, or rC/N-OMV in inducing CTLs, natural killer (NK) cells, and cytotoxic T cells to release granzyme B (*p* > 0.05).

## DISCUSSION

In order to develop an effective and safe vaccine against HCV, our previous study was evaluated with bioinformatic online tools and produced successfully a truncated recombinant fusion protein (rC/N) consisting of core (aa 1-118) and NS3 (aa 1095-1384), as two conserved parts of HCV, containing immunodominant epitopes[17]. In this study, the authors developed and verified *NM*B OMVs and used these microbial-derived

particles as an immunopotentiator to overcome the limitation of low immunogenicity of rC/N fusion protein vaccine. Analyses and evaluations in our study were performed by immunizing mice groups using rC/N alone or in mixed forms, with *NM*B OMVs, MF59, or Freund’s CIA adjuvants, and comparison were made with the control (OMVs, MF59, and PBS) groups.

The OMV is a reflective of the structure of the outer membrane and contains various compounds from the periplasm or cytoplasm without reproduction ability. Thus, detergent-generated vesicles are able to trigger the production of pro-inflammatory cytokines without harmful noxious effects[23-25,32]. The LAL test showed that the allowed endotoxin activity of LPS belonged to the prepared *NM*B OMVs (0.8 EU/ml), and they were considered safe; the authorized level of endotoxin activity for human use is less than 5 EU/ml. The absolute value of *NM*B OMVs’ zeta potential was more than 30 mV (38.1 > 30), indicating that the particles are in stable condition and are so small that they do not have the tendency to aggregate[35,36]. Typically, OMVs must be 50-300 nm in diameter. The zeta average of the achieved size [184.2 (d. nm)] matches the actual size of these particles[23,24,36]. A heterogeneity index or PDI (0.178) of less than 0.5 is an ideal indicator of uniform distribution of particles[35,36]. Thus, the physicochemical properties of OMVs indicated that they have potential to consider as adjuvants.

Cellular immune response, which is elicited by CTLs, is crucial for a therapeutic vaccine against HCV, but an ideal anti-HCV vaccine candidate (therapeutic and anaphylactic) should include CTLs, HTLs, and B-cells to ensure robust incitation of a humoral anti-HCV immune response[3,4]. Immunogenicity was monitored through the ability of splenic mononuclear cells to proliferate and induce Th1 (IFN-γ and IL-2), Th2 (IL-4), and Th17 (IL-17) cytokines. Furthermore, the concentrations of specific total antibody and isotypes, as indicators of Th2 (IgG1 and Th1 (IgG2a), were detected in the sera of immunized mice groups. The rates of increase in granzyme B in the granules of CTLs, NK cells, and cytotoxic T cells of the spleens of vaccinated mice groups were the indicators of apoptosis and cytotoxic activity[37,38].

The levels of total and isotype (IgG-1 and IgG-2a) immunoglobulins were significantly higher in the sera of mice immunized with rC/N-OMV compared to the control groups. The presence of IgG1 isotype is an indicator of Th2/humoral immune response, while IgG2a isotype is an indicator of Th1/cellular immune response[34]. The rate of isotypes (IgG1 and IgG2a) of antibodies showed that rC/N-OMV is able to induce humoral (Th2)- and cellular (Th1)-oriented immune responses. IFN-γ or type II interferon, is a cytokine that is critical for innate and adaptive immunity against pathogens. It has the ability to inhibit viral replication. This cytokine is produced predominantly by NK and NKT cells, by CD4 Th1 and CD8 CTL effector T cells and also by non-cytotoxic innate lymphoid cells[39]. Generally, IL-2, as a predominant cytokine that is produced during the primary response of Th cells, is required for the proliferation and differentiation of the Th precursor into functional Th cells, as well as the development of regulatory T cells. Thus, IL-2 is secreted by mature T cells to activate effector cells[40-42].

The levels of IFN-γ and IL-2 cytokines, as two arms of the cellular immune response in the spleens of the mice immunized with the candidate rC/N-OMV vaccine, amplified significantly in comparison with the control groups. Thus, the rC/N-OMV, as a safe candidate of vaccine, is able to strongly induce Th1-oriented (IgG2a antibody, IFN-γ, and IL-2 cytokines) responses. The data indicated that the administration of rC/N protein accompanied by *NM*B OMVs improved the Th2 polarized humoral-immune response as evidenced by increases in IL-4 cytokine and IgG1 antibody ratios that were less than that of rC/N-MF59.

Recently, the traditional paradigm, according to only Th1/Th2 balance, has been changed, and the distinct lineage of CD4 Th cell, called Th17, as the producer of effector molecules including IL-17, IL-17F, IL-21, IL-22, and IL-6 is undeniable[43]. The role of the Th17 cell lineage in the primary immune response has been documented, and it may be critical for vaccine-induced memory immune responses[43]. Researchers have proven that IL-17 induces protective cellular responses against several pathogens and is a connector between innate and adaptive immunity at the mucosa[44,45]. The results of the current study showed that the pro-inflammatory IL-17 producing lymphocytes in the spleens of mice immunized with the candidate rC/N-OMV vaccine amplified significantly compared with the OMV, PBS, and no antigen groups. In addition, although the presence of a protective immune response by IL-17 is evident, no significant difference was found among the groups vaccinated with rC/N-OMV, rC/N-Freund’s CIA, and rC/N-MF59. It seems that OMV, Freund’s CIA, and MF59 as adjuvants are equally able to induce IL-17.

The level of all cytokines (IFN -γ, IL-2, IL-4, and IL-17) in the spleen, and the total IgG and its isotypes (IgG1 and IgG2a) were higher in the seum of mice immunized with rC/N-OMVs than in the mice group vaccinated with rC/N alone. Therefore, *NM*B OMV showed its efficacy as an adjuvant beside the selected synthesized antigen (rC/N). It is noteworthy that the ratio of IFN-γ, IL-2, and IL-17 as Th1 indicators significantly increased in comparison with IL-4 (Th2 indicator) in the spleen of the mice immunized with rC/N-OMV, and in the same group, the rate of IgG2a (Th1 indicator) increased more than IgG1 (Th2 indicator). Although the candidate vaccine (rC/N-OMV) is able to induce both cellular and humoral immune responses, it seems that the cellular response is dominant.

The release of granzyme B, as enzymatic granules, mainly from CTLs, NK cells, and cytotoxic T cells is an indicator of apoptotic and cytotoxic activity[37,38]. The present study showed a significantly greater presence of granzyme B as cytotoxic granules in the spleens of mice immunized with rC/N-OMVs than in the control groups (*p* = 0.0001). Therefore, it could be concluded that the candidate vaccine is capable of activating the apoptosis of infected cells by CTLs and NKs.

The indicators show that the rC/N-OMV group is able to induce a greater Th1 (IFN-γ, IL-2, and IgG2a) immune response than the rC/N-MF59 group, and that *NM*B OMV is able to induce a greater Th1 response than MF59, which has been approved for use in humans. While rC/N-MF59 is capable of inducing Th2 (IL4 and IgG1) more than the other vaccinated groups, the rC/N-OMV group is able to compete with rC/N-Freund’s CIA immunized group. The highly effective Freund’s CIA is used as the gold standard to evaluate vaccines under development. Indeed, rC/N-*NM*B OMVs presented broad humoral and cellular immunity, and the cellular immune response was more robust than the humoral response. It also increased cytotoxicity by releasing granzyme B as apoptotic granules.

Taken together, the immunological benchmarks of rC/N together with the safety profile of *NM*B OMV as an immunoadjuvant are evidenced to consider this candidate vaccine as an effectual combination to produce remarkable anti-HCV responses.
